# Genome-Wide Interaction and Pathway Association Studies for Body Mass Index

**DOI:** 10.3389/fgene.2019.00404

**Published:** 2019-05-01

**Authors:** Hongxiao Jiao, Yong Zang, Miaomiao Zhang, Yuan Zhang, Yaogang Wang, Kai Wang, R. Arlen Price, Wei-Dong Li

**Affiliations:** ^1^Research Center of Basic Medical Sciences, Tianjin Medical University, Tianjin, China; ^2^Department of Genetics, College of Basic Medical Sciences, Tianjin Medical University, Tianjin, China; ^3^College of Public Health, Tianjin Medical University, Tianjin, China; ^4^Raymond G. Perelman Center for Cellular and Molecular Therapeutics, Children’s Hospital of Philadelphia, Philadelphia, PA, United States; ^5^Laboratory Medicine, Department of Pathology, University of Pennsylvania, Philadelphia, PA, United States; ^6^Department of Psychiatry, Center for Neurobiology and Behavior, University of Pennsylvania Perelman School of Medicine, Philadelphia, PA, United States

**Keywords:** epistasis, obesity, genome wide, pathway associations, *EXOC4*, *TOB1*

## Abstract

**Objective:** We investigated gene interactions (epistasis) for body mass index (BMI) in a European-American adult female cohort via genome-wide interaction analyses (GWIA) and pathway association analyses.

**Methods:** Genome-wide pairwise interaction analyses were carried out for BMI in 493 extremely obese cases (BMI > 35 kg/m^2^) and 537 never-overweight controls (BMI < 25 kg/m^2^). To further validate the results, specific SNPs were selected based on the GWIA results for haplotype-based association studies. Pathway-based association analyses were performed using a modified Gene Set Enrichment Algorithm (GSEA) (GenGen program) to further explore BMI-related pathways using our genome wide association study (GWAS) data set, GIANT, ENGAGE, and DIAGRAM Consortia.

**Results:** The *EXOC4*-1q23.1 interaction was associated with BMI, with the most significant epistasis between rs7800006 and rs10797020 (*P* = 2.63 × 10^-11^). In the pathway-based association analysis, Tob1 pathway showed the most significant association with BMI (empirical *P* < 0.001, FDR = 0.044, FWER = 0.040). These findings were further validated in different populations.

**Conclusion:** Genome-wide pairwise SNP-SNP interaction and pathway analyses suggest that EXOC4 and TOB1-related pathways may contribute to the development of obesity.

## Introduction

Obesity is a worldwide epidemic associated with increased morbidity of chronic diseases, including diabetes, cardiovascular diseases, metabolic syndrome, and cancer. In 2015, 603.7 million adults and 107.7 million children were obese; furthermore, in many countries the incidence of obesity continues to rise, doubling since 1980 (Afshin et al., 2017). This in turn imposes an enormous burden on the public health system. Many studies have shown that 40–70% of inter-individual variability in obesity can be attributed to genetic factors (Zaitlen et al., 2013; Locke et al., 2015). Currently, large-scale genome-wide association studies (GWASs) and meta-analyses have successfully identified in excess of 75 loci associated with obesity (Fall and Ingelsson, 2014). Nevertheless, these genetic variability can explain only a minor fraction of obesity cases (Li et al., 2010; Speliotes et al., 2010). This is partly due to the existence of other mechanisms such as epigenetics, gene-environment, and gene-gene interactions, that influence the heritability of obesity (Gibson, 2010; Wang X. et al., 2010). Almost one-third of the genetic variance in the etiology of obesity were due to non-additive factors, according to the family, twin and adoption studies (Stunkard et al., 1986; Price, 1987; Sorensen et al., 1989; Stunkard et al., 1990).

SNP-SNP interactions are considered to be potential sources of the unexplained heritability of common diseases (Manolio et al., 2009). In research to date on the influence of interactions, most studies invariably selected loci based on biological knowledge and known associated loci, studies of genome-wide gene x gene interactions are rare. Speliotes et al. (2010) tested SNP-SNP interaction effects among 32 BMI-associated SNPs in their GWAS result, however, no significant results were obtained after multiple test corrections. Young et al. (2016) found the interaction rs11847697(PRKD1)-rs9939609 (FTO) associated with BMI via pairwise SNP × SNP interactions analysis based on 34 established BMI-related SNPs in European American adolescents. Ding et al. (2012) also examined Gene-Gene interactions for abdominal obesity in Chinese population. Nevertheless, these studies ignored genomic regions that were not individually associated but could contribute to disease development if combined.

Until now, following the traditional GWAS approach, genome-wide interaction analyses (GWIAs) were used to investigate SNP-SNP interactions. This method did not need the selection of candidate sites, but computational time was a very large barrier. With the advancement of computing technology, the major barrier has been overcome, and SNP-SNP interaction studies gradually focused on the whole genome level. Wei et al. (2012) performed GWIAs for BMI using multiple human populations, and found eight interactions that had a significant *P*-value in one or more cohorts. Their studies further demonstrated the GWIA is an effective approach to explain the genetic factor of BMI. SNP-SNP interactions have always been explained by mapping to gene-gene interactions, and genome-wide pathway-based association analysis will further support the interpretation of gene-gene interactions. “Pathway” means a gene set collected from the same biological or functional pathway. Pathway-based association analysis will measure the correlations between phenotypes and gene sets based on the whole genome. This approach can provide additional biological insights and allow one to explore new candidate genes (Wang K. et al., 2010).

Compared to association analysis, fewer studies have assessed potential gene-gene interactions in obesity, and the relatively high heritability of obesity still has not been completely explained. We explored genome-wide IBD (identical by descent) sharing in obese families using linkage with data derived from genome-wide genotyping data, observing an interaction between 2p25-p24 and 13q13-21 that may influence extreme obesity (Dong et al., 2005). In the present study, we sought to discover novel susceptibility loci through assessing interaction effects with BMI across the whole genome, and to determine how multiple genetic variants contribute to the development of obesity.

## Materials and Methods

### Subjects

One thousand and seventy-one (1071) unrelated European American adults were recruited, 1030 of which were females. In this study, we carried out our analyses only in females, comprising 493 extremely obese cases (BMI > 35 kg/m^2^) and 537 never over-weight controls (BMI < 25 kg/m^2^). The collection processes have been described in our previous report (Wang et al., 2011). All participants gave informed consent, and the investigation protocol was approved by the Committee on Studies Involving Human Beings at the University of Pennsylvania.

### Genome-Wide Interaction Analysis

About 550,000 SNP markers were genotyped by Illumina HumanHap 550 SNP Arrays in our previous GWAS (Wang et al., 2011). PLINK 1.90 was used to perform GWIA for BMI. Due to the computational-demand, we used the “–fast-epistasis” command to screen for association. This test was based on a Z-score for the difference in SNP1-SNP2 association (odds ratio) between cases and controls by logistic regression, Z = [log(R)-log(S)]/sqrt[SE(R) + SE(S)], where R and S are the odds ratios in cases and controls, respectively (Purcell et al., 2007). We excluded SNPs of minor allele frequencies (MAF) < 5%. After frequency and genotyping pruning, 497174 SNPs were used to carry out interaction analyses. A total of 123,590,744,551 valid SNP-SNP tests were performed. We then selected the SNPs with interaction *P* < 1 × 10^-8^ (Bonferroni-corrected significant threshold *P* = 4.05 × 10^-13^) to analyze interactions by logistic regression based on allele dosage for each SNP.

### Haplotype-Based Association Analysis

Eight hundred and thirty-one (831) SNP-SNP interactions showed *P* < 10^-8^ in the results of the SNP-SNP interaction tests based on Z-scores. In order to rule out the possibility of an accidental finding, we mapped these SNPs to genes, then excluded the SNP-SNP interactions by the following criteria: ① neither SNPs exist in genes; ② either of the two SNPs exist independently in a gene. Through the above exclusion criteria, the rs7800006(*EXOC4*)-rs10797020(1q23.1) interaction was the most significant (*P* = 2.63 × 10^-11^), where there were 39 interactions with *P* < 10^-8^ between *EXOC4* and 1q23.1. Five SNPs exist in the *EXOC4* gene region and 9 SNPs exist in the 1q23.1 region, but their interaction *P*-value did not pass Bonferroni multiple tests. However, the Bonferroni correction test is highly conservative and would overcorrect for the non-independent SNPs, which fall within blocks of strong linkage disequilibrium (LD) (Duggal et al., 2008). Morris and Kaplan (2002) have reported that haplotype-based association analyses are more powerful than single allele-based methods when multiple disease-susceptibility mutations occur within the same gene. Epstein and Satten (2003) also have pointed out that haplotypes are useful during disease development due to the interaction of multiple cis-acting susceptibility variants located at the gene.

Therefore, in case of producing false negatives, we selected the 5 SNPs that exist in *EXOC4* and the 9 SNPs that exist in 1q23.1, respectively, for the next haplotype-based association analysis, which were conducted by PLINK1.07. The haplotype windows were defined at two SNPs, three SNPs, and four SNPs.

### Genome-Wide Pathway-Based Association Analysis

To further study the gene-gene interactions by pathway analysis, the GenGen program was used to analyze pathway-based association based on the modified Gene Set Enrichment Algorithm (GSEA) (Subramanian et al., 2005; Wang et al., 2007). The calculation steps have been outlined previously (Li et al., 2015). In this study, a total of 518230 SNPs passed the quality-control thresholds of minor allele frequencies > 0.01 and Hardy-Weinberg equilibrium > 0.001, which covered 17,438 genes, mapping SNPs to 20 kb upstream and downstream of each gene. A total of 1347 gene sets were selected from BioCarta, Kyoto Encyclopedia of Genes and Genomes (KEGG), and Gene Ontology (GO) databases, gene set sizes were between 5 and 200 genes.

### Replication of the Pathway-Based Association Results

We further attempted to replicate the GenGen results in data sets from the GIANT (*N* = 339,224) (Locke et al., 2015), ENGAGE (*N* = 87,048) (Horikoshi et al., 2015), and DIAGRAM (*N* = 119,688) (Wood et al., 2016) consortia. Given that no phenotypes and genotypes were available online from the three consortia, GSA-SNP software (Nam et al., 2010) was carried out to perform the pathway associations analyses using the GWAS *P*-values. To better compare with GenGen analysis results, we obtained SNP specific *P*-values from GIANT, ENGAGE, and DIAGRAM GWASs, and the same SNPs identified by the GenGen analysis were selected for the pathway association analysis for BMI in the three consortium data sets.

**Figure 1:**
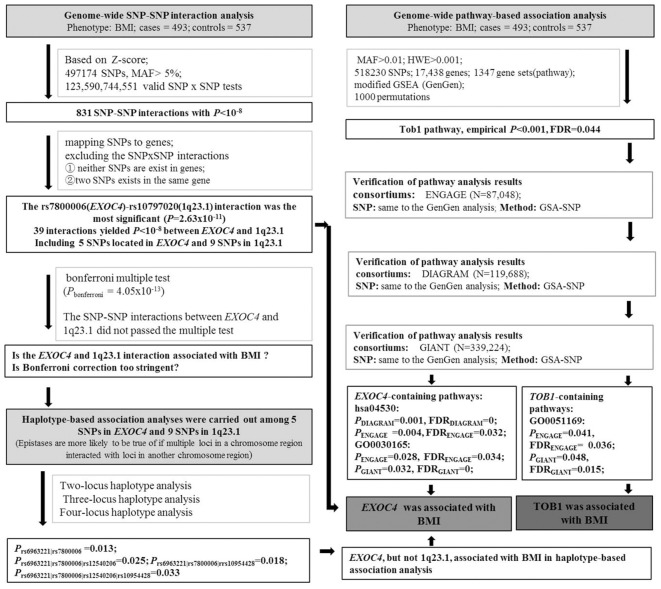
The flow chart of experimental analysis.

As described above, the flow chart of experimental analysis was shown in Figure 1.

## Results

**Figure 2:**
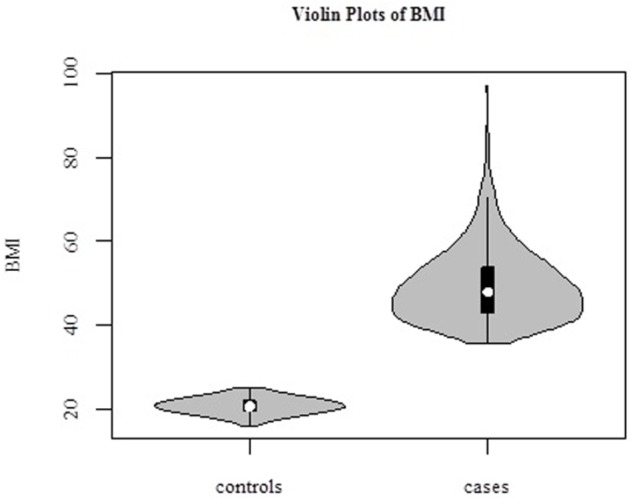
Violin plots of BMI in 1030 samples. Cases (BMI > 35 kg/m^2^, *N* = 493), and controls (BMI < 25 kg/m^2^, *N* = 537).

The average age of the 1030 female subjects was 42.2 ± 9.0 years (range, 17–65 years). In our study, we defined BMI > 35 kg/m^2^ as “cases,” *N* = 493, and BMI < 25 kg/m^2^ as “controls,” *N* = 537 (Figure 2). Distributions of BMI in cases and controls are shown in Table 1.

**Table 1:** BMI distributions in cases and controls.

	N	Age	BMI (kg/m^2^)	Maximum	Minimum	Mean	Std. Deviation
Cases	493	41.0 ± 9.2	>35.0	97.0	35.6	49.4	8.7
Controls	537	43.3 ± 8.6	<25.0	25.0	16.0	20.1	1.8

### Genome-Wide Interaction Analysis

**Figure 3:**
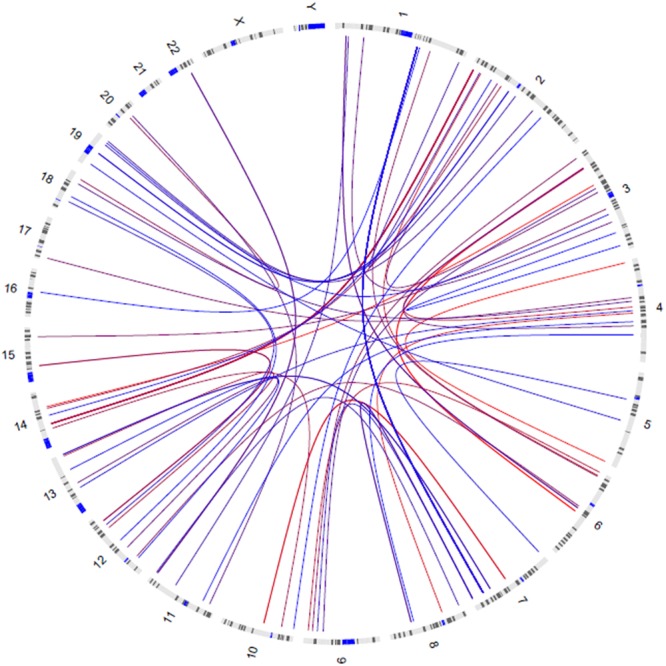
Circos visualization of mapped SNP-SNP interactions for BMI (*P* < 1 × 10^-9^). The curves represent the interactions between the two SNPs, and the color gradually changes from red to blue as the *P*-value decreases.

GWIA based on Z-score of BMI determined 831 SNP-SNP interactions with *P* < 10^-8^, those with *P* < 1 × 10^-9^ were shown in Figure 3. To avoid errors caused by chance and rare genotypes, some interactions were excluded according to the exclusion criteria, which has been described in method. rs7800006*(EXOC4*)-rs10797020(1q23.1) interaction yielded the lowest *P*-value (*P* = 2.63 × 10^-11^) after screening by exclusion criteria, but did not pass the threshold for multiple testing (*P* < 4.05 × 10^-13^). Fourteen SNPs resulted in 39 interactions that had *P* < 10^-8^ between *EXOC4* and 1q23.1. Five SNPs (rs10954428, rs12540206, rs6963221, rs7800006, and rs6976491) were found in the *EXOC4* gene region, while 9 SNPs (rs1578761, rs975118, rs10489833, rs10797020, rs11264997, rs7512592, rs6679056, rs1873511, and rs6697656) were found in 1q23.1among which the maximum distance is 70.4 kb (Table 2).

**Table 2:** Genotype interactions (epistasis) associated with BMI.

SNP1	MAF1	Gene1	SNP2	MAF2	Gene2	*P*-value^∗^ (allele dosage)	*P*-value^∗∗^ (Z-score)
rs10954428	0.339	*EXOC4*	rs10797020	0.449	Between OR10R1P and OR6Y1	4.24 × 10^-8^	1.44 × 10^-8^
rs10954428	0.339	*EXOC4*	rs1578761	0.452	Between OR10R1P and OR6Y1	2.04 × 10^-7^	8.66 × 10^-8^
rs10954428	0.339	*EXOC4*	rs10489833	0.450	Between OR10R1P and OR6Y1	6.25 × 10^-8^	2.26 × 10^-8^
rs10954428	0.339	*EXOC4*	rs11264997	0.452	Between OR10R1P and OR6Y1	8.54 × 10^-8^	3.21 × 10^-8^
rs12540206	0.424	*EXOC4*	rs1578761	0.452	Between OR10R1P and OR6Y1	4.95 × 10^-9^	1.21 × 10^-9^
rs12540206	0.424	*EXOC4*	rs975118	0.453	Between OR10R1P and OR6Y1	3.43 × 10^-9^	7.92 × 10^-10^
rs12540206	0.424	*EXOC4*	rs10489833	0.450	Between OR10R1P and OR6Y1	1.97 × 10^-9^	4.19 × 10^-10^
rs12540206	0.424	*EXOC4*	rs10797020	0.449	Between OR10R1P and OR6Y1	7.25 × 10^-10^	1.26 × 10^-10^
rs12540206	0.424	*EXOC4*	rs11264997	0.452	Between OR10R1P and OR6Y1	1.70 × 10^-9^	3.41 × 10^-10^
rs12540206	0.424	*EXOC4*	rs7512592	0.450	Between OR10R1P and OR6Y1	1.97 × 10^-9^	4.19 × 10^-10^
rs12540206	0.424	*EXOC4*	rs1873511	0.453	OR10R3P	4.38 × 10^-9^	1.04 × 10^-9^
rs12540206	0.424	*EXOC4*	rs6697656	0.452	Between OR6Y1 and OR6P1	1.13 × 10^-8^	3.28 × 10^-9^
rs6963221	0.413	*EXOC4*	rs1578761	0.452	Between OR10R1P and OR6Y1	9.53 × 10^-9^	2.67 × 10^-9^
rs6963221	0.413	*EXOC4*	rs975118	0.453	Between OR10R1P and OR6Y1	6.81 × 10^-9^	1.83 × 10^-9^
rs6963221	0.413	*EXOC4*	rs10489833	0.450	Between OR10R1P and OR6Y1	3.81 × 10^-9^	9.33 × 10^-10^
rs6963221	0.413	*EXOC4*	rs10797020	0.449	Between OR10R1P and OR6Y1	1.43 × 10^-9^	2.87 × 10^-10^
rs6963221	0.413	*EXOC4*	rs11264997	0.452	Between OR10R1P and OR6Y1	2.40 × 10^-9^	5.29 × 10^-10^
rs6963221	0.413	*EXOC4*	rs7512592	0.450	Between OR10R1P and OR6Y1	3.81 × 10^-9^	9.33 × 10^-10^
rs6963221	0.413	*EXOC4*	rs6679056	0.471	OR10R2	2.58 × 10^-8^	8.63 × 10^-9^
rs6963221	0.413	*EXOC4*	rs1873511	0.453	OR10R3P	6.21 × 10^-9^	1.61 × 10^-9^
rs6963221	0.413	*EXOC4*	rs6697656	0.452	Between OR6Y1 and OR6P1	2.11 × 10^-8^	6.83 × 10^-9^
rs6976491	0.423	*EXOC4*	rs1578761	0.452	Between OR10R1P and OR6Y1	7.46 × 10^-9^	2.01 × 10^-9^
rs6976491	0.423	*EXOC4*	rs975118	0.453	Between OR10R1P and OR6Y1	5.11 × 10^-9^	1.31 × 10^-9^
rs6976491	0.423	*EXOC4*	rs10489833	0.450	Between OR10R1P and OR6Y1	3.00 × 10^-9^	7.00 × 10^-10^
rs6976491	0.423	*EXOC4*	rs10797020	0.449	Between OR10R1P and OR6Y1	1.13 × 10^-9^	2.16 × 10^-10^
rs6976491	0.423	*EXOC4*	rs11264997	0.452	Between OR10R1P and OR6Y1	1.89 × 10^-9^	4.01 × 10^-10^
rs6976491	0.423	*EXOC4*	rs7512592	0.450	Between OR10R1P OR6Y1	3.00 × 10^-9^	7.00 × 10^-10^
rs6976491	0.423	*EXOC4*	rs6679056	0.471	OR10R2	2.09 × 10^-8^	7.03 × 10^-9^
rs6976491	0.423	*EXOC4*	rs1873511	0.453	OR10R3P	4.81 × 10^-9^	1.20 × 10^-9^
rs6976491	0.423	*EXOC4*	rs6697656	0.452	Between OR6Y1 and OR6P1	1.68 × 10^-8^	5.30 × 10^-9^
rs7800006	0.427	*EXOC4*	rs1578761	0.452	Between OR10R1P and OR6Y1	1.31 × 10^-9^	2.65 × 10^-10^
rs7800006	0.427	*EXOC4*	rs975118	0.453	Between OR10R1P and OR6Y1	8.72 × 10^-10^	1.66 × 10^-10^
rs7800006	0.427	*EXOC4*	rs10489833	0.447	Between OR10R1P and OR6Y1	5.26 × 10^-10^	9.00 × 10^-11^
rs7800006	0.427	*EXOC4*	rs10797020	0.449	Between OR10R1P and OR6Y1	1.96 × 10^-10^	2.63 × 10^-11^
rs7800006	0.427	*EXOC4*	rs11264997	0.452	Between OR10R1P and OR6Y1	4.42 × 10^-10^	7.09 × 10^-11^
rs7800006	0.427	*EXOC4*	rs7512592	0.450	Between OR10R1P and OR6Y1	5.26 × 10^-10^	9.00 × 10^-11^
rs7800006	0.427	*EXOC4*	rs6679056	0.471	OR10R2	6.87 × 10^-9^	1.98 × 10^-9^
rs7800006	0.427	*EXOC4*	rs1873511	0.453	OR10R3P	1.12 × 10^-9^	2.19 × 10^-10^
rs7800006	0.427	*EXOC4*	rs6697656	0.452	Between OR6Y1 and OR6P1	3.10 × 10^-9^	7.41 × 10^-10^

**^∗^***Interaction analysis based on allele dosage for each SNP.*

**^∗∗^***Interaction analysis based on Z-score.*

*MAF, minor allele frequency.*

### Haplotype-Based Association Analysis

Due to the highly conservative of Bonferroni correction test, false negatives were prone. We selected the above-mentioned 14 SNPs located in *EXOC4* or 1q23.1 for haplotype-based association analyses. The SNPs showed LD in both *EXOC4* (D’ > 0.94), and 1q23.1 (D’ > 0.99) (Supplement Figure 1). Two-locus haplotype analysis revealed that rs6963221| rs7800006 (A| C) was associated with BMI (*P* = 0.013). BMI was also influenced by three-locus haplotypes rs6963221| rs7800006| rs12540206 (A|C|date, fewer studies have examined T, *P* = 0.025), rs6963221| rs7800006| rs10954428 (A|C|G, *P* = 0.018) and the four-locus haplotype rs6963221| rs7800006| rs12540206| rs10954428 (A|C|T|G, *P* = 0.033) (Table 3). The four SNPs are in *EXOC4*, indicating that *EXOC4* associated with BMI.

**Table 3:** Haplotype analysis of *EXOC4* gene SNPs.

SNPs	Haplotype	F_A^∗^	F_U^∗∗^	χ^2^	DF	*P*
rs6963221| rs7800006	AC	0.036	0.015	6.146	1	0.013
rs6963221| rs7800006| rs12540206	ACT	0.032	0.014	5.026	1	0.025
rs6963221| rs7800006| rs10954428	ACG	0.033	0.014	5.624	1	0.018
rs6963221| rs7800006| rs12540206| rs10954428	ACTG	0.029	0.013	4.559	1	0.033

**^∗^***Frequency in cases.*

**^∗∗^***Frequency in controls.*

### Genome-Wide Pathway-Based Association Analysis

**Figure 4:**
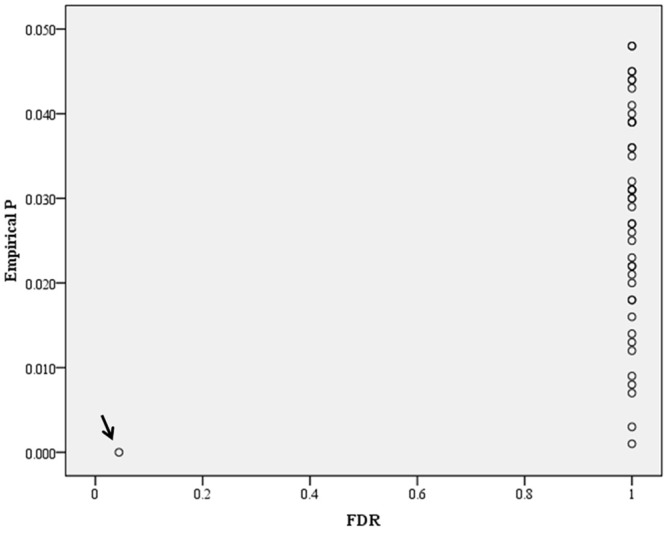
Distribution of empirical *P*-FDR for BMI. Empirical *P*-FDR for BMI- related pathways (empirical *P* < 0.05, denoted as “nominal” *P*-values in the GenGen program) obtained by modified GSEA (GenGen), *Tob1* pathway is indicated by the arrow.

In the genome-wide pathway-based association study carried out with GenGen, 43 pathways achieved a significance of empirical *P* < 0.05 (Figure 4 and Supplement Table 1). The Tob1 pathway (role of Tob in T-cell activation) showed the most significant association with BMI (empirical *P* < 0.001, FDR = 0.044, FWER = 0.040, Table 4). Empirical *P*-values (denoted as “nominal *P*” values by the GenGen program) were calculated based on the 1000 phenotype permutations.

**Table 4:** Pathway-based association study for BMI.

Pathway ID	Gene set	Consortium	Empirical *P*	*P*	Corrected-*P*^∗^	FDR^∗∗^	FWER^∗∗∗^	Method
tob1Pathway	Role of Tob in T-cell activation	Our-data	<0.001			0.044	0.040	GenGen
GO0051169	Nuclear	GIANT		0.048	0.622	0.015		GSA-SNP
	transport	ENGAGE		0.041	0.329	0.036		GSA-SNP
hsa04530	Tight	ENGAGE		0.004	0.487	0.032		GSA-SNP
	junction	DIAGRAM		0.001	0.277	0		GSA-SNP
GO0030165	PDZ	GIANT		0.032		0		GSA-SNP
	domain binding	ENGAGE		0.028		0.034		GSA-SNP

**^∗^***Benjamini and Hochbaum false discovery rate (Benjamini and Hochberg, 1995).*

**^∗∗^***False discovery rate used by Subramanian et al. (2005).*

**^∗∗∗^***Family wise-error rate.*

Replication studies were conducted in data sets from the GIANT, ENGAGE, and DIAGRAM consortia by GSA-SNP. The Tob1 pathway did not have a significant *P*-value in these settings. However, the pathway GO0051169 (nuclear transport) containing TOB1 was associated with BMI in GIANT and ENGAGE consortia, and passed FDR correction for multiple testing (*P*_GIANT_ = 0.048, FDR_GIANT_ = 0.015; *P*_ENGAGE_ = 0.041, FDR_ENGAGE_ = 0.036, Table 4). The EXOC4-contained pathway hsa04530 was also associated with BMI in ENGAGE and DIAGRAM consortium data sets by GSA-SNP (Table 4). GO0030165 containing *EXOC4* was also related to BMI in the GIANT and ENGAGE consortium data sets and passed FDR correction (Table 4).

## Discussion

In the context of genetic epidemiology, although GWASs have found the majority of BMI-related genes identified to date, combined these loci explain only about 4% of the phenotypic variation of BMI (Loos, 2018). Modest and rare variants have been ignored by the GWASs, partly because of the other mechanisms, including epigenetics, gene-gene and gene-environment interactions, and statistical issues (Gibson, 2010; Wang X. et al., 2010; Chiefari et al., 2013, 2016; Lee et al., 2014). To date, fewer studies have examined the effects of interactions on obesity. Despite this, some obesity-related interactions still have been found, including *PRKD1*-*FTO* and *WNT4*-*WNT5A* (Wei et al., 2012; Young et al., 2016; Dong et al., 2017). Pathway-based analysis is an alternative approach to detect gene interactions. Liu et al. (2010) had found that the vasoactive intestinal peptide pathway was significantly correlated with BMI and fat mass, suggesting that this pathway plays an important role in the development of obesity. Our previous studies also revealed that the Rac1pathway was associated with the obesity-related phenotype plasma adiponectin (Li et al., 2015).

In the present study, our GWIA for BMI found an interaction between *EXOC4* and 1q23.1 that may contribute to the development of obesity, although this interaction did not pass the Bonferroni correction test, they had the lowest interaction *P*-value (*P* = 4.05 × 10^-13^) after accidental exclusion. To further examine whether *EXOC4* and 1q23.1 were related to BMI, we selected the SNPs locate in *EXOC4* and 1q23.1 accordingly base on the results of GWIA to carry out haplotype-based association analyses, the results verified that *EXOC4* contributed to BMI. In genome-wide pathway-based association studies, the relation between the *TOB1* pathway and BMI was identified. *EXOC4* and *TOB1* associated with BMI were replicated in GIANT, ENGAGE, and DIAGRAM data sets. To our knowledge, these findings have not been identified having main effects in previous BMI-related studies.

*EXOC4* (exocyst complex component 4, also known as *SEC8*) is a component of the exocyst complex involved in the targeting of exocytic vesicles, which participate in temporal and spatial regulation of exocytosis (Hsu et al., 1996; TerBush et al., 1996). Numerous research results show that exocysts interact directly or indirectly with many proteins including cell membranes, cytoskeletal, the small GTPases and other proteins in the cell cortex (Wu et al., 2008; Tanaka and Iino, 2015). Tanaka et al. indicated that *EXOC4* modulates cell migration by controlling the *ERK* and p38 *MAPK* signaling pathways (Tanaka and Iino, 2015). They also found that *EXOC4* can mediate cell migration and adhesion via controlling Smad3/4 expression through CBP (Tanaka et al., 2017).

*EXOC4* is located in a widely replicated obesity linkage peak on chromosome 7q22-q36 (Feitosa et al., 2002; Li et al., 2003), and has been connected with various diseases, such as type 2 diabetes, cancer, and neuronal disorders. GLUT4 (glucose transporter 4) transports most of the glucose in muscle and adipose tissue; the docking and tethering of the GLUT4 vesicle to the plasma membrane is mediated via *EXOC4* (Inoue et al., 2003; Inoue et al., 2006). A population genetic study also identified several type 2 diabetes-associated SNPs near *EXOC4* in The NHLBI Family Heart Study (Laramie et al., 2008).

Nineteen genes are involved in BMI-related Tob1 pathway (role of Tob in T-cell activation): *TOB1, TOB2, IFNG, IL2, IL2RA, IL4, SMAD3, SMAD4, TGFB1, TGFB2, TGFB3, TGFBR1, TGFBR2, TGFBR3, CD3D, CD3E, CD3G, CD247*, and *CD28*. This pathway is a component of balanced functioning of the immune system. TOB1 represses T cell activation and is a member of a family of genes with anti-proliferative properties. Research has shown that TOB1 interacts with the TGF (transforming growth factor) and can stimulate transcription factors *SMAD4* and *SMAD2*, increasing their binding to the *IL-2* promoter and helping to repress *IL-2* expression, suggesting that interference in TOB1 function be associated with autoimmune disease (Tzachanis et al., 2001; Tzachanis and Boussiotis, 2009; Gibson, 2010). Numerous studies have found a significant correlation between obesity and many autoimmune diseases, adipokines such as leptin, adiponectin and resistin may be key players in interactions among them (Versini et al., 2014).

TOB1and TOB2 belong to the TOB family of anti-proliferative proteins that have the potential to regulate cell growth. As a repressor of the p38/*MAPK* pathway, *TOB1* can suppress p38/*MAPK* signaling by decreasing phosphorylation of p38 and *ATF2* (Sun et al., 2013; Ng et al., 2017); p38/*MAPK* acts as an enhancer of adipogenesis contributes to obesity (Patel et al., 2003). The miR-32-*TOB1*-*FGF21* pathway can regulate brown adipose tissue adipocyte function and development and is associated with obesity and metabolic syndrome (Ng et al., 2017). The biological functions mentioned above are consistent with our study results and provided evidence of a direct connection between *TOB1* and obesity.

Traditional GWASs have identified many obesity-associated genes, however, additional loci have yet to be identified. *EXOC4* and *Tob1* pathway genes may be among these from our GWIA and genome-wide pathway-based association analysis.

EXOC4 join in the tight junction signal pathway: this pathway receives not only assembly signals but also transmit information (Zihni et al., 2014). Therefore, EXOC4 may play a role in signal transmission from sensory perception to the brain, thus affecting obesity. The Tob1 pathway may contribute to obesity through the MAPK pathway. Needless to say, molecular biological experiments are needed to repeat the results. For the GWASs, statistical replication is the golden rule to prevent false positives. Although our findings were replicated in different populations with different methods, it also needs to be confirmed in larger populations by GWIAs.

## Ethics Statement

All participants gave informed consent, and the investigation protocol was approved by the Committee on Studies Involving Human Beings at the University of Pennsylvania.

## Author Contributions

W-DL designed the study, researched data, and edited the manuscript. HJ researched data and wrote the manuscript. KW researched data and edited the manuscript. RP designed the study and contributed to the discussion. YoZ, MZ, and YuZ researched data. YW researched data and contributed to discussion. All authors have reviewed the manuscript.

## Conflict of Interest Statement

The authors declare that the research was conducted in the absence of any commercial or financial relationships that could be construed as a potential conflict of interest.
