# Supplementary material for: Genome-Wide Interaction and Pathway Association Studies for Body Mass Index
Source: Front Genet. 2019 May 1;10:404. doi: 10.3389/fgene.2019.00404 (PMC6504780; doi:10.3389/fgene.2019.00404)
Supplement: Supplementary file 1 [file Table_1.DOC]

**Supplement**

**Jiao et al. Genome-wide Interaction and Pathway Association Studies for Body Mass Index**

**Supplement Table1** Pathway-based association study for BMI by GenGen in extremely obese cases (N=493) and never-overweight controls (N=537) (Nominal *P*<0.05)

| **Pathway ID** | **Set Size** | **ES** | **NES** | **Nominal *P*** | **FDR** | **FWER** | **Database** |
| --- | --- | --- | --- | --- | --- | --- | --- |
| tob1Pathway | 19 | 0.682 | 3.332 | 0 | 0.044 | 0.04 | BioCarta |
| GO0014812 | 8 | 0.776 | 2.465 | 0.001 | 1 | 0.954 | GO |
| GO0015399 | 99 | 0.433 | 2.594 | 0.003 | 1 | 0.882 | GO |
| GO0016776 | 29 | 0.553 | 2.495 | 0.007 | 1 | 0.94 | GO |
| GO0031532 | 9 | 0.661 | 1.593 | 0.008 | 1 | 1 | GO |
| GO0016820 | 101 | 0.424 | 2.412 | 0.009 | 1 | 0.966 | GO |
| stathminPathway | 18 | 0.565 | 1.909 | 0.012 | 1 | 0.924 | BioCarta |
| CSKPathway | 21 | 0.536 | 2.071 | 0.013 | 1 | 0.84 | BioCarta |
| GO0016862 | 10 | 0.633 | 1.614 | 0.014 | 1 | 1 | GO |
| GO0048704 | 9 | 0.706 | 2.006 | 0.016 | 1 | 1 | GO |
| skp2e2fPathway | 10 | 0.573 | 1.211 | 0.018 | 1 | 1 | BioCarta |
| GO0048488 | 6 | 0.747 | 1.609 | 0.018 | 1 | 1 | GO |
| GO0048705 | 12 | 0.656 | 2.076 | 0.02 | 1 | 0.999 | GO |
| GO0009267 | 18 | 0.504 | 1.435 | 0.021 | 1 | 1 | GO |
| GO0005158 | 19 | 0.641 | 1.995 | 0.022 | 1 | 1 | GO |
| hsa05340 | 31 | 0.475 | 2.017 | 0.022 | 1 | 0.924 | KEGG |
| GO0042135 | 6 | 0.689 | 1.383 | 0.023 | 1 | 1 | GO |
| GO0005160 | 8 | 0.64 | 1.292 | 0.025 | 1 | 1 | GO |
| il17Pathway | 15 | 0.563 | 1.706 | 0.026 | 1 | 0.98 | BioCarta |
| akapCentrosomePathway | 15 | 0.574 | 1.907 | 0.027 | 1 | 0.924 | BioCarta |
| GO0043393 | 15 | 0.598 | 2.023 | 0.027 | 1 | 1 | GO |
| GO0016653 | 7 | 0.599 | 1.154 | 0.029 | 1 | 1 | GO |
| GO0009225 | 12 | 0.585 | 1.869 | 0.03 | 1 | 1 | GO |
| GO0032365 | 8 | 0.647 | 1.385 | 0.03 | 1 | 1 | GO |
| fibrinolysisPathway | 12 | 0.569 | 1.34 | 0.031 | 1 | 1 | BioCarta |
| fbw7Pathway | 9 | 0.586 | 1.179 | 0.031 | 1 | 1 | BioCarta |
| GO0001578 | 7 | 0.653 | 1.298 | 0.031 | 1 | 1 | GO |
| pelp1Pathway | 7 | 0.657 | 1.266 | 0.032 | 1 | 1 | BioCarta |
| GO0005984 | 5 | 0.651 | 1.109 | 0.035 | 1 | 1 | GO |
| GO0031579 | 5 | 0.745 | 1.512 | 0.036 | 1 | 1 | GO |
| GO0031623 | 5 | 0.719 | 1.368 | 0.036 | 1 | 1 | GO |
| GO0045060 | 5 | 0.724 | 1.405 | 0.039 | 1 | 1 | GO |
| GO0019894 | 9 | 0.569 | 1.112 | 0.039 | 1 | 1 | GO |
| hsa05040 | 32 | 0.42 | 1.684 | 0.039 | 1 | 0.992 | KEGG |
| plcePathway | 11 | 0.605 | 1.731 | 0.04 | 1 | 0.98 | BioCarta |
| tcraPathway | 11 | 0.627 | 1.66 | 0.041 | 1 | 0.983 | BioCarta |
| GO0046942 | 58 | 0.439 | 1.737 | 0.043 | 1 | 1 | GO |
| GO0005123 | 7 | 0.559 | 1.093 | 0.044 | 1 | 1 | GO |
| hsa00600 | 36 | 0.445 | 1.697 | 0.044 | 1 | 0.992 | KEGG |
| GO0046627 | 5 | 0.728 | 1.361 | 0.045 | 1 | 1 | GO |
| GO0044269 | 5 | 0.653 | 1.206 | 0.045 | 1 | 1 | GO |
| plcdPathway | 5 | 0.771 | 1.669 | 0.048 | 1 | 0.983 | BioCarta |
| mitochondriaPathway | 19 | 0.451 | 1.215 | 0.048 | 1 | 1 | BioCarta |
