# Supplementary figures and images for: Genome-Wide Interaction and Pathway Association Studies for Body Mass Index
Source: Front Genet. 2019 May 1;10:404. doi: 10.3389/fgene.2019.00404 (PMC6504780; doi:10.3389/fgene.2019.00404)

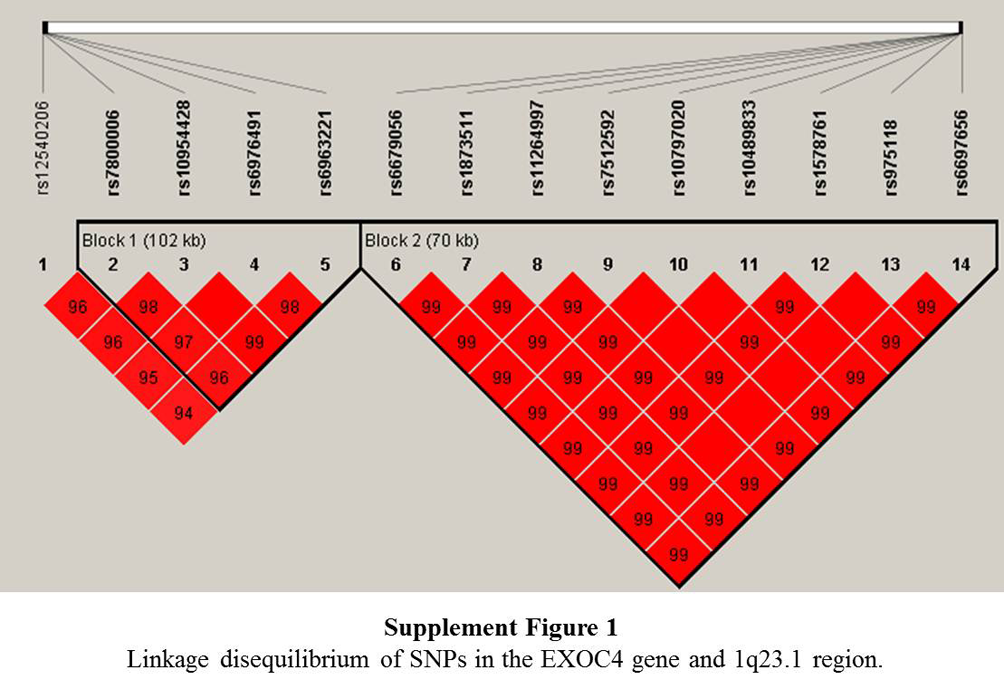

Supplement: Supplementary file 2 [file Image_1.TIF]
